# Differential Gender Effects in the Relationship between Perceived Immune Functioning and Autistic Traits

**DOI:** 10.3390/ijerph14040409

**Published:** 2017-04-12

**Authors:** Marlou Mackus, Deborah de Kruijff, Leila S. Otten, Aletta D. Kraneveld, Johan Garssen, Joris C. Verster

**Affiliations:** 1 Division of Pharmacology, Utrecht University, 3584 CG Utrecht, The Netherlands; m.mackus@uu.nl (M.M.); deborahdekruijff@gmail.com (D.d.K.); l.s.otten@students.uu.nl (L.S.O.); a.d.kraneveld@uu.nl (A.D.K.); j.garssen@uu.nl (J.G.); 2 Institute for Risk Assessment Sciences (IRAS), Utrecht University, 3584 CS Utrecht, The Netherlands; 3 Nutricia Research, 3584 CT Utrecht, The Netherlands; 4 Centre for Human Psychopharmacology, Swinburne University, Melbourne 3122, Australia

**Keywords:** autism, immune functioning, gender

## Abstract

Altered immune functioning has been demonstrated in individuals with autism spectrum disorder (ASD). The current study explores the relationship between perceived immune functioning and experiencing ASD traits in healthy young adults. N = 410 students from Utrecht University completed a survey on immune functioning and autistic traits. In addition to a 1-item perceived immune functioning rating, the Immune Function Questionnaire (IFQ) was completed to assess perceived immune functioning. The Dutch translation of the Autism-Spectrum Quotient (AQ) was completed to examine variation in autistic traits, including the domains “social insights and behavior”, “difficulties with change”, “communication”, “phantasy and imagination”, and “detail orientation”. The 1-item perceived immune functioning score did not significantly correlate with the total AQ score. However, a significant negative correlation was found between perceived immune functioning and the AQ subscale “difficulties with change” (r = −0.119, *p* = 0.019). In women, 1-item perceived immune functioning correlated significantly with the AQ subscales “difficulties with change” (r = −0.149, *p* = 0.029) and “communication” (r = −0.145, *p* = 0.032). In men, none of the AQ subscales significantly correlated with 1-item perceived immune functioning. In conclusion, a modest relationship between perceived immune functioning and several autistic traits was found.

## 1. Introduction

Autism spectrum disorder (ASD) is a neurodevelopmental disease, characterized by an inability to form normal social relationships or communication and restricted and repetitive behavior [1]. The prevalence of ASD is four times higher in men than in women [2].

Anomalies of the immune system are frequently described among a subset of individuals with ASD, and are often associated with the primary features of ASD [3,4,5]. These include neuroinflammation, the presence of auto-antibodies, and increased T cell responses. Additionally, enhanced innate natural killer cells (NKCs) and monocyte immune responses have been reported in ASD individuals [5]. Such dysfunctions in the immune system are suggested to directly interfere with neurodevelopment and neurological processes that are causing changes in behavior.

Not solely the functioning of the immune system of the child may affect neurological development, active maternal infections as well as aberrant immune response of the mother during pregnancy are seemingly important with regard to prenatal risks in developing ASD [6]. Next to infections, in about 20% of mothers with children at risk for developing ASD, maternally derived auto-antibodies primed against particular fetal brain proteins were found [4]. These maternal auto-antibodies can cross the placenta and reach the fetal brain. They are known to recognize seven developmentally brain proteins in the fetal brain, contributing to the stereotypical behaviors of an autistic individual [7].

In general, it is not as well understood how cytokine signaling mediates these immunological changes affecting neurodevelopment. An imbalance between T regulatory cells and T effector cells, however, is identified. A particular imbalance increases vulnerability to pathogens, as a result of a less active immune response after encounter. Immune functioning can be said to be lowered, and in turn there is increased risk of inflammation. In addition, imbalance in regulatory T cells causes tolerance to certain antigens to be reduced or even absent, leading to the development of autoimmune diseases [8]. Besides development of autoimmune diseases, increased Immunoglobulin-E (IgE) as a result of T regulatory imbalances results in increased IgE-mediated allergies, frequently reported in autistic children compared to healthy controls [9,10].

Together, these effects on specific immune cells demonstrate impaired immune functioning in autistic individuals, causing them to be more vulnerable to the development of infectious diseases or immune diseases and perceiving reduced immune functioning. The aim of the current study was to examine the association between perceived immune functioning and the presence of autism spectrum traits in healthy young adults.

## 2. Materials and Methods

Students of Utrecht University, The Netherlands, 18 to 30 years old, were recruited to complete a survey. Informed consent was obtained from all participants; no formal ethics approval was required to conduct this survey according to the Central Committee of Research Involving Human Subjects, the Netherlands.

Demographic data was collected, including data on smoking, alcohol consumption, and drug use. The Autism Spectrum Quotient (AQ) was completed to examine variations in autistic traits in individuals with normal intelligence [11]. The AQ is a quantitative self-test and instrument for assessing the degree to which a person with normal intelligence expresses traits associated ASD. The scale consists of 50 items assessing personal preferences and habits. Subjects must rate to what extent they agree or disagree with statements on a Likert scale. The four answer categories are “definitely agree”, “slightly agree”, “slightly disagree”, and “definitely disagree”. The item scores are summed, with a high total score corresponding to a high autistic load. The AQ has 5 subscales: “social insights and behavior”, “difficulties with change”, “communication”, “phantasy and imagination”, and “detail orientation”.

Perceived immune functioning was examined with the Immune Function Questionnaire (IFQ), a scale assessing immunological aspects related to lowered immunity [12]. The IFQ assesses the frequency of various symptoms associated with poor immune functioning. As a direct relation to immune impairments, 18 symptom items were included in the questionnaire: sore throat, headaches, flu, runny nose, coughing, cold sores, boils, mild fever, warts, pneumonia, bronchitis, sinusitis, sudden high fever, ear infection, diarrhea, meningitis, eye infection, sepsis, and long healing injuries (due to a translation error, the item “boils” was omitted from the analyses). These symptoms were rated on a 5-point Likert-type scale, having to choose from “never”, “once or twice”, “occasionally”, “regularly” and “frequently,” with scores from 0 to 4, respectively. Participants were asked to rate these symptoms as experienced in the previous 12 months. A high total score reflects poor immune functioning.

Perceived immune functioning and general health was also scored as a single-item question that could be scored from 0 (very poor) to 10 (excellent) [13]. Subject could further indicate whether they perceived having reduced immune functioning. A previous study showed that IFQ scores correlated significantly with the 1-item perceived immune functioning score [14].

IBM SPSS statistics version 24 (IBM, North Castle, NY, USA) was used for data analysis. Total AQ score, and its subscale scores, were correlated to the total IFQ score, and the 1-item perceived health and immune functioning rating, applying nonparametric Spearman’s rho correlations. Analyses were conducted for the whole sample, and for men and women separately. Participants reporting mental illness or currently using psychoactive medication were excluded from the analyses.

## 3. Results

N = 410 students participated in the study (55.6% women), having a mean (SD) age of 20.5 (2.3) years old. Overall, participants graded their mean (SD) health with a 7.8 (0.9), and their mean (SD) immune functioning with a 7.9 (1.1). The mean (SD) IFQ score was 11.5 (5.7).

Table 1 summarizes the descriptive characteristics of the whole sample, and for men and women separately.

Total IFQ score significantly correlated with the 1-item scores of perceived general health (r = −0.283, *p* = 0.000), and perceived immune functioning (r = −0.450, *p* = 0.000). No significant gender differences were found for the immune functioning outcomes. Men did score significantly higher on the total AQ than women (*p* = 0.005). A similar result was found for the subscales “social insights and behavior” (*p* = 0.013), “communication” (*p* = 0.032), and “phantasy and imagination” (*p* = 0.001).

The 1-item perceived immune functioning score did not significantly correlate with the total AQ score. However, a significant negative correlation was found between perceived immune functioning and the AQ subscales “difficulties with attention” (r = −0.119, *p* = 0.019).

In women, 1-item perceived immune functioning correlated significantly with the AQ subscales “difficulties with change” (r = −0.149, *p* = 0.029) and “communication” (r = −0.145, *p* = 0.032). In men, none of the subscales significantly correlated with 1-item perceived immune functioning.

Total IFQ scores significantly positively correlated with the AQ subscales “difficulties with change” (r = 0.152, *p* = 0.003) and “communication” (r = 0.126, *p* = 0.013), however, no significant correlation was revealed with the total AQ score. In women, the total IFQ score significantly correlated with the subscales “difficulties with change” (r = 0.160, *p* = 0.020) and “communication” (r = 0.167, *p* = 0.014). In men, the total IFQ correlated significantly with the total AQ score (r = 0.159, *p* = 0.039), and the subscales “difficulties with change” (r = 0.205, *p* = 0.007) and “communication” (r = 0.155, *p* = 0.040).

Perceived general health significantly negatively correlated with the total AQ score (r = −0.107, *p* = 0.036), and the subscales “difficulties with change” (r = −0.137, *p* = 0.007) and “communication” (r = −0.153, *p* = 0.002). In women, significant negative correlations were found between perceived general health and the AQ subscales “difficulties with change” (r = −0.165, *p* = 0.015) and “communication” (r = −0.136, *p* = 0.043). In men, perceived general health significantly negatively correlated only with the AQ subscale “communication” (r = −0.209, *p* = 0.005).

In women, the same AQ subscales significantly negatively correlated with perceived immune functioning (r = −0.136, *p* = 0.045 and r = −0.148, *p* = 0.028 respectively). In men, none of the subscales significantly correlated with perceived immune functioning.

Total IFQ scores significantly positively correlated with the AQ subscales “attention” (r = 0.128, *p* = 0.011) and “communication” (r = 0.110, *p* = 0.028); however, no significant correlation was revealed with the total AQ score. In women, the total IFQ score significantly correlated with the subscale “communication” (r = 0.145, *p* = 0.031) only. In men, in contrast, the total IFQ correlated significantly with the total AQ score (r = 0.153, *p* = 0.046), as well as the subscales “attention” (r = 0.206, *p* = 0.006) “communication” (r = 0.148, *p* = 0.049).

## 4. Discussion

Overall, the autistics traits of having “difficulties with change” and “communication” deficits were significantly associated with poorer perceived immune functioning. However, clear gender differences were observed in this study. In woman, the subscales “difficulties with change” and “communication” deficits correlated significantly with the 1-item perceived immune functioning score. In men, these correlations were not significant.

The observed gender differences may suggest a role of sex hormones in the development of autistic traits. Testosterone seems to be the relevant link between ADS and immune functioning, because of its possible immunosuppressing function [15,16]. Studies suggest that physiological concentrations of the female sex hormones stimulate immune functioning, whereas those of the male sex hormone testosterone suppresses [17].

The influence of sex hormones on immune functioning is established early in development, starting already in utero [18]. The normal process of brain masculinization is mediated by cells and signaling molecules that are related to inflammation, modified by exposure to high levels of testosterone. The innate immune system together with inflammatory signaling molecules seem to be directing brain masculinization, for men being more vulnerable to internal and external inflammatory mediators [19]. Studies on gene expression profiles in ASD males revealed that the genes identified were largely involved in astrocyte and microglia activation [20]. In addition, overactivation of the neuroimmune system has been associated with the risk of developing ASD. Together, these observations suggest that inflammatory mediators may function as fundamental regulators of male brain development [21,22], and that the natural process of brain masculinization may put males at a risk of higher vulnerability for inflammation during critical periods of brain development. This may explain why the prevalence of ASD is higher in men than in women.

Autistic traits can be viewed as exaggerated masculine behaviors, which may explain the higher autism spectrum quotient score in men. However, as 1-item perceived immune functioning scores in men were not significantly associated with AQ subscale scores, the current findings do not support the hypothesis that testosterone suppresses immune functioning. Given this, it is likely that other factors such as genetic predisposition also influence the differential development of autistic traits in men and women. The latter may also be explained by the fact that men naturally have more prenatal testosterone, and are more masculine than women.

Finally, some limitations need to be addressed. In the current study we excluded people with mental illness, including ASD. Hence, the sample consists of healthy young participants. This should be taken into account when interpreting the data. The magnitude of the observed differences in scores on perceived immune functioning and autistic trait scales between men and women was small (see Table 1), and its importance can be debated. It is however interesting to see that, even in a healthy sample, significant associations were found between perceived immune functioning and ASD traits, including modest but significant gender differences. In a patient population, it can be expected that these associations are even stronger. Future research should investigate this. A limitation of the current study was that we did not include objective assessments of immune functioning but relied on subjective measures such as the IFQ. For the future use of these questionnaires it is important to demonstrate their relationship with objective assessments of immune functioning such as changes in cytokine levels in blood or saliva. It is important to keep in mind that subjective reports of the immune system may not completely correspond with objective measures. For instance, individuals with autistic traits may be more observant concerning their health status, or report more symptoms without being more ill. Future research should address these issues.

## 5. Conclusions

Taken together, the modest but significant correlations observed in this study further support the potential link between having autism traits and reduced immune functioning, although this seems to be gender-dependent. Further research, preferably in a patient population, is required to elucidate these differential gender effects.

## Figures and Tables

**Table 1:** Descriptives.

	Women	Men	All Participants
	N = 228	N = 182	N = 410
**Male/Female ratio**	55.6%	44.4%	44.4%/55.6%
**Age**	20.2 (2.2)	20.8 (2.4)	20.5 (2.3)
**Height (m)**	1.69 (0.07)	1.84 (0.07) *	1.75 (0.09)
**Weight (kg)**	62.7 (9.1)	74.7 (10.0) *	68.1 (11.2)
**Perceived health**	7.7 (0.9)	7.9 (0.9)	7.8 (0.9)
**Perceived immune functioning**	7.8 (1.1)	8.0 (1.2) *	7.9 (1.2)
**Total IFQ score**	11.6 (5.1)	9.9 (5.3) *	10.8 (5.3)
**Total AQ score**	99.9 (13.1)	105.1 (12.8) *	102.2 (13.2)

Abbreviations: IFQ: Immune Function Questionnaire; AQ: Autism-Spectrum Quotient. Significant gender differences (*p* < 0.05) are indicated by *.

## References

[1] Jyonouchi H., Sun S., Itokazu N. (2002). Innate immunity associated with inflammatory responses and cytokine production against common dietary proteins in patients with autism spectrum disorder. Neuropsychobiology.

[2] Werling D.M., Geschwind D.H. (2013). Sex differences in autism spectrum disorders. Curr. Opin. Neurol..

[3] Nardone S., Elliot E. (2016). The interaction between the immune system and epigenetics in etiology of autism spectrum disorders. Front. Neurosci..

[4] Meltzer A., van de Water J. (2017). The role of the immune system in autism spectrum disorder. Neuropsychopharmacology.

[5] Mead J., Ashwood P. (2015). Evidence of supporting an altered immune response in ASD. Immunol. Lett..

[6] Shi L., Fatemi S.H., Sidwell R.W., Patterson P.H. (2003). Maternal influenza infection causes marked behavioral pharmacological changes in the offspring. J. Neurosci..

[7] Braunschweig D., Krakowiak P., Duncason P., Boyce R., Hansen R.L., Ashwood P., Hertz-Picciotto I., Pessah I.N., Van de Water J. (2013). Autism-specific maternal autoantibodies recognize critical proteins in developing brain. Transl. Psychiatry.

[8] Enstrom A.M., Van de Water J.A., Ashwood P. (2009). Autoimmunity in autism. Curr. Opin. Investig. Drugs.

[9] Magalhaes E.S., Pinto-Mariz F., Bastos-Pinto S., Pontes A.T., Prado E.A., de Azevedo L.C. (2009). Immune allergic response in Asperger Syndrome. J. Neuroimmunol..

[10] Gurney J.G., McPheeters M.L., Davis M.M. (2006). Parental report of health conditions and health care use among children with and without autism: National Survey of Children’s Health. Arch. Pediatr. Adolesc. Med..

[11] Hoekstra R.A., Bartels M., Cath D.C., Boomsma D.I. (2008). Factor structure, reliability and criterion validity of the Autism Spectrum Quotient (AQ): A study in Dutch population and patient groups. J. Autism Dev. Disord..

[12] Reed P., Vile R., Osborne L.A., Romano M., Truzoli R. (2015). Problematic internet usage and immune function. PLoS ONE.

[13] Donners A.A., Tromp M.D., Garssen J., Roth T., Verster J.C. (2015). Perceived immune status and sleep: A survey among Dutch students. Sleep Disord..

[14] Van Schrojenstein Lantman M., Otten L.S., Mackus M., de Kruijff D., van de Loo A.J.A.E., Kraneveld A.D., Garssen J., Verster J.C. (2017). Mental resilience, perceived health and immune status. J. Multidiscip. Healthc..

[15] Wichmann M.W., Zellweger R., DeMaso C.M., Ayala A., Chaudry I.H. (1996). Mechanism of immunosuppression in males following trauma-hemorrhage. Critical role of testosterone. Arch. Surg..

[16] Wichmann M.W., Angele M.K., Ayala A., Cioffi W.G., Chaudry I.H. (1997). Flutamide: A novel agent for restoring the depressed cell-mediated immunity following soft-tissue trauma and hemorrhagic shock. Shock.

[17] Grossman C.J. (1984). Regulation of the immune system by sex steroids. Endocr. Rev..

[18] McCarthy M.M., Wright C.L. (2017). Convergence of sex differences and the neuroimmune system in autism spectrum disorder. Biol. Psychiatry.

[19] McCarthy M.M., Pickett L.A., VanRyzin J.W., Kight K.E. (2015). Surprising origins of sex differences in the brain. Horm. Behav..

[20] Werling D.M., Parikshak N.N., Geschwind D.H. (2016). Gene expression in human brain implicates sexually dimorphic pathways in autism spectrum disorders. Nat. Commun..

[21] McCarthy M.M. (2008). Estradiol and the developing brain. Physiol. Rev..

[22] McCarthy M.M., De Vries G., Forger N., Pfaff D., Arnold A.P., Etgen A.M., Fahrbach S.E., Rubin R.T. (2009). Sexual differentiation of the brain: Mode, mechanisms and meaning. Hormones, Brain and Behavior.

